# Initial reports of the SARS‐CoV‐2 Delta variant (B.1.617.2 lineage) in Bangladeshi patients: Risks of cross‐border transmission from India

**DOI:** 10.1002/hsr2.366

**Published:** 2021-09-08

**Authors:** Md. Shazid Hasan, Md. Tanvir Islam, A. S. M. Rubayet Ul Alam, Shovon Lal Sarkar, M. Shaminur Rahman, Ovinu Kibria Islam, Md. Ali Ahsan Setu, Tanay Chakrovarty, Hassan M. Al‐Emran, Iqbal Kabir Jahid, M. Anwar Hossain

**Affiliations:** ^1^ Department of Microbiology Jashore University of Science and Technology Jashore Bangladesh; ^2^ Department of Microbiology University of Dhaka Dhaka Bangladesh; ^3^ Department of Biomedical Engineering Jashore University of Science and Technology Jashore Bangladesh; ^4^ Genome Center Jashore University of Science and Technology Jashore Bangladesh; ^5^ Vice Chancellor Jashore University of Science and Technology Jashore Bangladesh

To the Editor,

Severe acute respiratory syndrome coronavirus‐2 (SARS‐CoV‐2) has emerged as a global pandemic, causing 3.5 million deaths until May 1, 2021.^1^, ^2^, ^3^ The resurgence of the new mutating variants has led to an increase in the number of infections exacerbating the death toll in several countries of the world, making vaccination strategies unworthy.^4^, ^5^, ^6^, ^7^ World health organization (WHO) declares several variants as the “variant of concern” and Indian variants (Delta variants) are one of them.^8^ In India, the second wave started in March 2021, and by the end of April, they became the first country to report 400 000 cases every 24 hours, and the emerged new variants have played as key infectious agents.^9^ As of May 5, 2021, India has 20 282 833 confirmed cases with a death toll of 222 408.^10^ Due to the unprecedented rise in COVID‐19 cases, many countries have imposed a travel ban to and from India to stop the spread of the new variants.^11^ Bangladesh also closed its border for 14 days from April 25, 2021 and extended the border closure for another 2 weeks on May 8, 2021 due to the surge in COVID‐19 cases in India.^12^, ^13^ Bangladesh, as a neighboring country of India, initiated the monitoring of SARS‐CoV‐2 variants among the travelers returning from India. The Government of Bangladesh (GoB) was imposing the policy of institutional quarantine of 14 days. All travelers were screened for COVID‐19 diagnostic test by RT‐PCR, and the positive cases were screened SARS‐CoV‐2 variants at the Genome Centre of Jashore University of Science and Technology (GC, JUST). On May 6, a total of the first 16 samples of the travelers returning from India were tested, in which three samples were found positive by RT‐PCR using a commercial kit from Sansure Biotech Co., Ltd (China).

All three positive samples were amplified for the screening of the spike receptor‐binding domain (RBD) portion of the genome. PCR was performed with 7 μL of RNA sample using the Luna Universal One‐Step RT‐qPCR Kit (New England Biolabs Inc., Ipswich, Massachusetts) with the primers (Forward: F2: GCTGTAGACTGTGCACTTGACCC, Reverse: R1: CTCAGTAAGAACACCTGTGCC) for the spike portion of the genome (Supplementary method). The amplicons were later sequenced using the BigDye Terminator v3.1 Cycle Sequencing Kit (SeqStudio, Applied Biosystem, ThemoFisher Scientific, Inc., USA) as presented in supplementary method. The identification of the sequence as designated viral lineage was performed as shown in supplementary method. For revealing the complete genome data of the two sequenced samples, we approached the Ion S5 System using barcoded IonAmpliSeq libraries (Supplementary method). The Ethical Review Committee of JUST approved this investigation (ERC approval no: Ref/ERC/FBST/JUST/2020‐41). All participants were informed about the study objectives and its procedure before data collection. Data collectors collected written consents from all respondents, maintaining a physical distance.

A 17‐year‐old male accompanied his mother to travel to Kolkata, India for medical assistance and they were tested COVID‐19 negative. During their 107 days stay in Kolkata, they had rented a hotel room in a hotel and visited 2 days at Shova hospital, Kolkata. Another 40‐year‐old female patient went to India for the treatment of her waist pain on March 7, 2021, with her husband. They took COVID‐19 test on March 4, 2021, and with COVID‐19 negative they entered India and stayed at their relative's house in Bashirhat, Kolkata where they went to “Bashirhat District Hospital. She visited the hospital two times regarding her X‐ray of the waist and stayed around 48 days in her relatives' house before returning from India.

After their return from India, the male patient was diagnosed COVID‐19 positive on May 6, 2021 with no severe casualties, whereas the female patient was diagnosed COVID‐19 positive on April 25, 2021, with fever, coughing, vomiting, short breathing, and transient amnesia. She was prescribed O_2_ inhalation and bronchodilator medicine for breathing problem and painkillers for waist pain.

Both patients were recommended to perform erythrocyte sedimentation rate (ESR), complete blood count (CBC), S. creatinine, D‐Dimer test, HRCT of the lung, SGPT, and chest X‐ray, which were mandatory for a COVID‐19 patient (Table 1). However, due to financial constrain, some tests were not performed by them.

**TABLE 1:** Case 1 and Case 2 medical report of the male and female patient

Case 1: Medical report of the male patient	
Blood group	
ABO	B
Rh	Positive
Hematological report	
HGB (Hemoglobin) (cyanomethanoglobin method)	13.0 g/dL
ESR (westergren)	35 mm
Differential count WBC	
Total count of WBC	5300/cmm
Neutrophils	43%
Lymphocytes	48%
Eosinophils	3%
Monocytes	6%
Basophils	0%
Total count of platelet	210 000/cmm
Biochemical analysis report	
Random plasma glucose	5.8 mmol/L
S. Creatinine	0.9 mg/dL
SGPT (ALT)	32 U/L
Immunology report	
D‐Dimer	0.21

Case 2: Medical report of the female patient	
Hematological report	
HGB (hemoglobin) (cyanomethanoglobin method)	11.5 g/dL
ESR (westergren)	20 mm
Differential count WBC	
Total count of WBC	8200/cmm
Neutrophils	57%
Lymphocytes	35%
Eosinophils	6%
Monocytes	2%
Basophils	0%
Total count of platelet	1,86 000/cmm
Biochemical analysis report	
S. Bilirubin	0.8 mg/dL
SGPT (ALT)	32 U/L
SGOT (AST)	45
Immunology report	
D‐Dimer	Not done

Partial sequencing of the RBD portion of the spike protein revealed two featured mutations (L452R and T478K) of the B.1.617.2 PANGO lineage for both samples.^14^ Whole‐genome sequences revealed 38 mutations and 29 aa substitutions for the sample JUST1 (Collected from the female patient, GISAID accession ID—EPI_ISL_ 2 036 272) and 33 mutations and 26 aa substitutions for sample JUST2 (Collected from the male patient, GISAID accession ID—EPI_ISL_1942249). Genome blast in CoVsurver: Mutation analysis of hCoV‐19 (https://www.gisaid.org/epiflu-applications/covsurver-mutations-app/) showed that JUST1 and JUST2 have 99.93% and 99.35% genome identity, respectively, with three strains from West Bengal, India (WB1, WB2, and WB3) that were submitted in GISAID on May 12, 2021 (Accession‐EPI_ISL_2036277, EPI_ISL_2036278, EPI_ISL_2036279) (Figure 1A). A heat map prepared with amino acid substitutions was found in JUST1, JUST2, WB1, WB3, reference B.1.167.1 (GISAID accession ID—EPI_ISL_1372093), and reference B.1.167.2 (GISAID accession ID—EPI_ISL_2131509) strains (Figure 1B). The heat map revealed that 21 aa substitutions (including six spike protein substitutions) were common among JUST, WB, and B.1.167.2 samples. On the other hand, only nine aa substitutions (including four spike substitutions) found in reference B.1.167.1 strain were also found in the JUST, WB, and B.1.167.2 strains. Four other aa substitutions (N:T362I, ORF1a:P309L, ORF1a:H2092Y, and ORF7a:L116F) were found in JUST and WB strains, which were not found in reference B.1.167.1 and B.1.167.2 strains. Signature spike substitution S:E484Q found in B.1.167.2 was not present in any of the JUST and WB samples. Genome analysis indicated that the samples we analyzed contain SARS‐CoV‐2 strains that are closely related to the strains found in West Bengal, which correlate with the travel history of the patients. These data also confirm that both male and female patients were infected with the B.1.167.2 variant of the virus. The data also represent the molecular evidence of the patients' infections from West Bengal India. Finally, the Nextstrain server (https://nextstrain.org/sars-cov-2/) for SARS‐CoV‐2 phylodynamics also bolstered our interpretation based on the mutation pattern of the strains.

**FIGURE 1:**
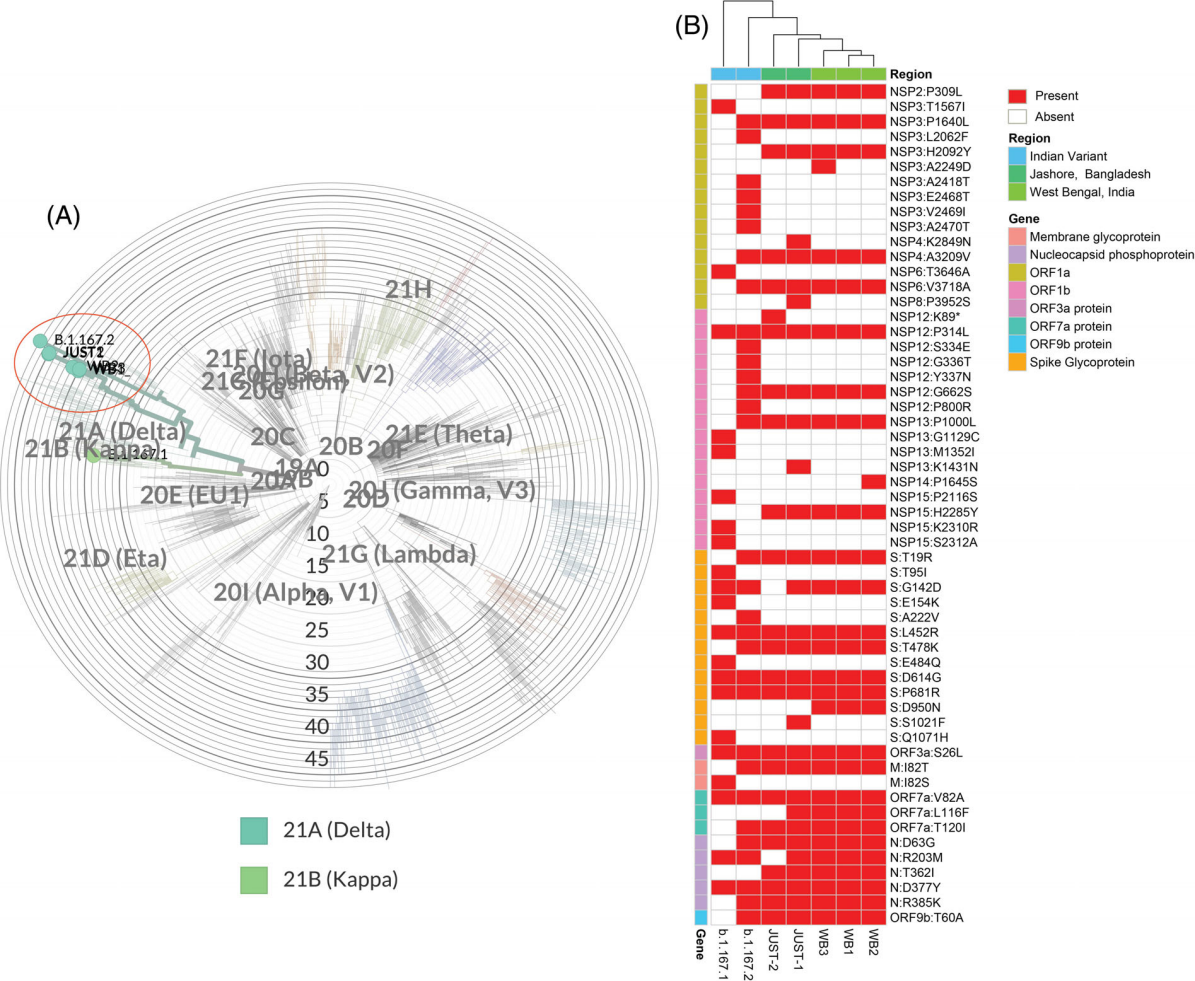
Comparison of amino acid substitutions retrieved from genome sequences of seven SARS‐CoV‐2 viruses. A, Phylogenetic tree of SARS‐CoV‐2 uses five isolates (JUST1, JUST2, WB1, WB2, WB3 and two references [B.1.167.1 and B.1.167.2 strains]). The study isolates were highlighted with circle. The tree was condensed using Nextclade (https://clades.nextstrain.org/). B, Amino acid substitutions found in our study samples (JUST1, JUST2) are compared with three West Bengal (WB1, WB2, WB3) and reference B.1.167.1 and reference B.1.167.2 strains. Mutations and amino acid substitutions were retrieved from Nextclade (https://clades.nextstrain.org/)

The southwest part of Bangladesh is adjacent to India, which had on‐road passage between these countries and can be a potential route of transmission of SARS‐CoV‐2 through returning passengers. Indian population was receiving the Oxford‐Astrazeneca vaccine, although there was a resurgence of South African (Beta variant) and Indian variants (Delat variants). The Delta variants were 50% more transmissible and 60% more lethal (For every one death 1.6 death in ratio compared with the previous version of the virus) with reduced affinity for neutralizing antibody (The L452R mutation confers a reduction of the recognition capability of the immune system) as demonstrated by other studies.^15^, ^16^ Transmission of these new variants may cause a similar impact among Bangladeshi population, which introduced the Oxford‐Astrazeneca vaccine. However, AstraZeneca can provide a 60% effectiveness against the variant in the population after 2 week from taking the second dose.^17^ The people returning from “India” must be kept under strict monitoring by sequencing the spike portion of any positive sample to counteract the expansion of these new variants in Bangladesh, especially the “Delta variants.” The report conclusively proves that without institutional monitoring, Bangladesh is under big threat to Delta variants like Nepal and United Kingdom.

## CONFLICTS OF INTEREST

The authors of this manuscript declare that they have no conflict of interest.

## AUTHOR CONTRIBUTIONS

Conceptualization: Hassan M. Al‐Emran, Iqbal Kabir Jahid.

Data Curation: Md. Shazid Hasan, Md. Tanvir Islam, Tanay Chakrovarty, Md. Ali Ahsan Setu, Shovon Lal Sarkar.

Formal Analysis: A. S. M. Rubayet Ul Alam, M. Shaminur Rahman, Ovinu Kibria Islam.

Investigation: Md. Shazid Hasan, Md. Tanvir Islam, Tanay Chakrovarty, Md. Ali Ahsan Setu, Shovon Lal Sarkar.

Methodology: Md. Shazid Hasan, Md. Tanvir Islam, Tanay Chakrovarty, Md. Ali Ahsan Setu, Shovon Lal Sarkar.

Project Administration: M. Anwar Hossain.

Writing – Original Draft Preparation: Md. Tanvir Islam, Md. Shazid Hasan, A. S. M. Rubayet Ul Alam, Ovinu Kibria Islam.

Writing – Review & Editing: Iqbal Kabir Jahid, M. Anwar Hossain.

All authors have read and approved the final version of the manuscript.

## Supporting information

**Data S1.** Supporting Information.

## Data Availability

The genomes under this study are available at GIDAID under accession no. EPI_ISL_ 2 036 272 and EPI_ISL_1942249.
