# Supplementary material for: Initial reports of the SARS‐CoV‐2 Delta variant (B.1.617.2 lineage) in Bangladeshi patients: Risks of cross‐border transmission from India
Source: Health Sci Rep. 2021 Sep 8;4(3):e366. doi: 10.1002/hsr2.366 (PMC8425784; doi:10.1002/hsr2.366)
Supplement: Supplementary file 1 — Data S1. Supporting Information. [file HSR2-4-e366-s001.docx]

**Initial reports of the SARS-CoV-2 Indian B.1.617. 2 lineage in Bangladeshi patients: risks of cross-border leakage/transmission from India**

Md. Shazid Hasan^1#^, Md. Tanvir Islam^1^#, A. S. M. Rubayet Ul Alam^1^, Shovon Lal Sarkar^1^, M. Shaminur Rahman^2^, Ovinu Kibria Islam^1^, Md. Ali Ahsan Setu^1^, Tanay Chakrovarty^1^, Hassan M. Al-Emran^3^, Iqbal Kabir Jahid^1,4^, M. Anwar Hossain^2,4,5,*^

1 Department of Microbiology, Jashore University of Science and Technology, Jashore-7408, Bangladesh

2 Department of Microbiology, University of Dhaka, Dhaka-1000, Bangladesh

3 Department of Biomedical Engineering, Jashore University of Science and Technology, Jashore-7408, Bangladesh

4 Genome Center, Jashore University of Science and Technology, Jashore-7408, Bangladesh

5 Vice-Chancellor, Jashore University of Science and Technology, Jashore-7408, Bangladesh

#Equal Contribution

***Corresponding author**

Prof. M. Anwar Hossain: hossaina@du.ac.bd

**Table 1: Luna one step PCR**

| **PCR mix** |
| --- |
| Reaction mix : 10ul  RT-enzyme mix : 1ul  S_F : 0.8ul  S_R : 1.2ul  Template : 7ul |
|  |
|  |
| **PCR conditions** |
| Temperature Time |
| RT 55^0^ C 12 min  Initial denaturation 95^0^ C 1 min |
| **Cycling part (40 cycles):** |
| Denaturation 95^0^ C 30 sec  Annealing 58^0^ C 45 sec  Extension 60^0^ C 1 min |
|  |
| Final extension 60^0^ C 7.5 min  Hold 4^0^ C ∞ |
|  |
|  |
| **Gel Run** |
| Gel concentration : 1% agarose  Gel volume : 60ul  Dye : Ethidium Bromide |
|  |
| Ladder : 50bp , 6-7ul /well  PCR product : 5ul/well |
|  |
| Voltage : 80 V  Current : 150 mA  Time : 40 min |

| **ExoSAP cleaning** |
| --- |
|  |
| ExoSAP IT : 1 ul  PCR product : 2.5 ul |
|  |
| **Incubation** |
| Temperature Time |
| Enzyme activation 37^0^C 15 min  Enzyme deactivation 80^0^C 15 min  Hold 4^0^C ∞ |
|  |
|  |
|  |
| **Cycle sequencing** |
|  |
| Big dye : 0.5 ul  5x buffer : 1.75 ul  Primer (S_R1) : 0.5 ul  H_2_O : 4ul  Product : 3.25 ul |
|  |
|  |
| **Sequencing PCR conditions** |
| Temperature Time |
| Initial denaturation 96^0^ C 1 min |
| **25 cycles** |
| Denaturation 96^0^ C 0.10 sec  Annealing 58^0^ C 0.05 sec |
|  |
| Extension 60^0^ C 2 min 30 seconds  Hold 4^0^ C ∞ |
|  |
| **SAM purification** |
| SAM solution : 45 ul  X terminator : 10 ul  Product : 10 ul  Shaking : 1500 rpm for 45 minutes  Centrifuge : 1000 x g for 2 minutes |

**Table 2: Sanger Sequencing protocol**

**Supplementary method:**

The sequenced FASTA files were cleaned with Chromas Pro (https://technelysium.com.au/wp/) and aligned with the reference sequence (NC_045512.2/SARS-CoV-2/Wuhan-Hu-1) using MolecularEvolutionary Genetics Analysis (MEGA X) software (Kumar et al., 2018) to detect the presence of any mutations in spike region.

Viral RNAs were extracted from 140µL of leftover nasopharyngeal samples and eluted with 60 µL Buffer AVE (RNase-free water that contains 0.04% sodium azide to prevent microbial growthand subsequent contamination with RNases) provided with the kit (QIAamp Viral RNA Mini Kit). First-strand cDNAs were prepared from the extracted viral RNA using SuperScript™ III First-Strand Synthesis System (Invitrogen™, Thermo Fisher Scientific, USA). The concentrations of cDNA were determined using the dsDNA HS Assay Kit with Qubit 4 Fluorometer (Thermo Fisher Scientific, USA). The Ion AmpliSeq™ SARS‑CoV‑2 Research Panel (Thermo Fisher Scientific, USA) was used to prepare IonAmpliSeq™ libraries from previously prepared cDNA. Two 5X primer pools that target 237 amplicons specific to theSARS‑CoV‑2 were used during target amplification PCR and the number of amplification cycles was determined based on their viral copy number. Each amplified sample was subjected to 2µLFuPa reagents for partial digestion. Digested amplicons were ligated with Ion P1 adapter and Ion Xpress™ Barcode Adapters 1–16 kit (Ion Torrent™, Thermo Fisher Scientific, USA).Magnetic bead cleanup was performed using Agencourt™ AMPure™ XP Reagent (Beckman Coulter, USA) to purify barcoded IonAmpliSeq™ libraries.IonLibrary TaqMan® Quantitation Kit was used to determine the concentration of each Ion AmpliSeq™ library by qPCRfollowing the manufacturer’s instructions.The final concentrations of the libraries were diluted into 100 picomolar (pM) according to the manufacturer’s protocol. Allequimolar libraries were pooled for the preparation of template-positive Ion Sphere™ Particles (ISPs) using the Ion 520™ & Ion 530™ Kit – OT2 (Thermo Fisher Scientific, USA) on the Ion One Touch™ 2 System. Template-positive ISPs were enriched on the Ion One Touch™ ES system. The enriched template-positive ISPs along with control ISPs were loaded in Ion 520/530™ chip and sequenced in Ion S5™ System.
